# Disease-specific and glucocorticoid-responsive serum biomarkers for Duchenne Muscular Dystrophy

**DOI:** 10.1038/s41598-019-48548-9

**Published:** 2019-08-21

**Authors:** Yetrib Hathout, Chen Liang, Michael Ogundele, Ganggang Xu, Shefa M. Tawalbeh, Utkarsh J Dang, Eric P. Hoffman, Heather Gordish-Dressman, Laurie S. Conklin, John N. van den Anker, Paula R. Clemens, Jean K. Mah, Erik Henricson, Craig McDonald

**Affiliations:** 1 School of Pharmacy and Pharmaceutical Sciences, Binghamton University, Binghamton, NY 13902 USA; 2 Department of Mathematical Sciences, Binghamton University, Binghamton, NY 13902 USA; 3 Department of Biomedical Engineering, Binghamton University, Binghamton, NY 13902 USA; 4 Department of Management Science, University of Miami, Coral Gables, FL 33146 USA; 5 Division of Biostatistics, Children’s National Health System, Washington, DC 20010 USA; 6 Division of Gastroenterology, Children’s National Health System, Washington, DC 20010 USA; 7 Division of Clinical Pharmacology, Children’s National Health System, Washington, DC 20010 USA; 8 Neurology Service, Department of Veterans Affairs Medical Center, Department of Neurology, University of Pittsburgh, Pittsburgh, PA USA; 9 Department of Pediatrics, Cumming School of Medicine, University of Calgary, Alberta Children’s Hospital, Calgary, AB T3B 6A8 Canada; 10 Department of Physical Medicine & Rehabilitation, University of California, Davis School of Medicine, Davis, CA 95618 USA

**Keywords:** Prognostic markers, Proteomics

## Abstract

Extensive biomarker discoveries for DMD have occurred in the past 7 years, and a vast array of these biomarkers were confirmed in independent cohorts and across different laboratories. In these previous studies, glucocorticoids and age were two major confounding variables. In this new study, using SomaScan technology and focusing on a subset of young DMD patients who were not yet treated with glucocorticoids, we identified 108 elevated and 70 decreased proteins in DMD relative to age matched healthy controls (p value < 0.05 after adjusting for multiple testing). The majority of the elevated proteins were muscle centric followed by cell adhesion, extracellular matrix proteins and a few pro-inflammatory proteins. The majority of decreased proteins were of cell adhesion, however, some had to do with cell differentiation and growth factors. Subsequent treatment of this group of DMD patients with glucocorticoids affected two major groups of pharmacodynamic biomarkers. The first group consisted of 80 serum proteins that were not associated with DMD and either decreased or increased following treatment with glucocorticoids, and therefore were reflective of a broader effect of glucocorticoids. The second group consisted of 17 serum proteins that were associated with DMD and these tended to normalize under treatment, thus reflecting physiologic effects of glucocorticoid treatment in DMD. In summary, we have identified a variety of circulating protein biomarkers that reflect the complex nature of DMD pathogenesis and response to glucocorticoids.

## Introduction

Major advances have occurred in the past few years in the discovery of serum molecular biomarkers in patients affected with Duchenne muscular dystrophy (DMD). These include blood circulating proteins^1–3^, circulating miRNA^4–6^ and circulating metabolites^7,8^. Several of these biomarkers were confirmed using independent DMD cohorts and across different laboratories, and in animal models such as mdx mouse^9,10^ and golden retriever muscular dystrophy dog^11,12^, further confirming the association of these molecular biomarkers with dystrophin deficiency and dystrophic muscle pathogenesis. These DMD associated biomarkers, especially protein circulating biomarkers, can be grouped into four major categories which include muscle injury biomarkers or creatine kinase (e.g. CK like biomarkers), extracellular matrix remodeling biomarkers, muscle degeneration/regeneration associated factors as well as some inflammation and immune associated biomarkers^2,3^.

Efforts in bridging these serum circulating biomarkers to disease progression and response to therapies have been undertaken. Initial studies conducted in the mdx mouse model have shown that certain muscle injury biomarkers return to normal concentrations when dystrophin expression is restored in the skeletal muscle following treatment with peptide-antisense oligonucleotide conjugate^9,13^ but this finding remains to be tested and/or validated in DMD patients receiving dystrophin replacement therapies. Certain miRNAs also responded to dystrophin replacement therapies in animal models^6^ and were shown in a preliminary study using a small DMD patient cohort to correlate with functional performance^4^. Interestingly, glucocorticoids (GC), drugs commonly used to treat DMD, engaged a completely different set of biomarkers in both DMD patients^14^ and in the mdx mouse model^15^. GC mostly decreased the concentrations of several circulating pro-inflammatory and immune associated proteins as well as a large number of cell adhesion proteins, extracellular remodeling proteins and proteins associated with metabolism, but did not correct the concentrations of muscle injury biomarkers.

What we have learned from these previous studies is that age and GC use are two major confounding variables in defining reliable biomarkers for DMD. Hence, the focus of the present study is to refine the list of serum protein biomarkers using a subset of GC-naïve DMD patients (never treated with GC), classify these biomarkers in the context of muscle pathogenesis, and examine the effect of age and GC on the trajectory of these biomarkers in young DMD boys (4–10 years old).

## Results

### Cross-sectional biomarker analysis in GC-naïve DMD versus healthy controls

**Figure 1:**
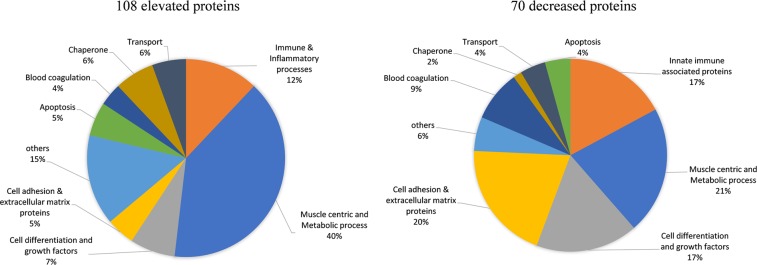
Pie chart depicting the biological process and molecular function of serum protein biomarkers identified in GC-naïve DMD (n = 18) compared to healthy controls (n = 12). The elevated proteins (108 in total) were mostly of muscle origin followed by some pro-inflammatory and cell adhesion proteins while the decreased proteins (70 in total) consisted mostly of cell adhesion and cell differentiation factors and some metabolism associated and innate immunity-associated proteins.

We sought to identify robust disease associated biomarkers in DMD, while specifically accounting for confounding of age and GC use on biomarker levels. In this new analysis, a cohort of 31 young DMD patients (4–10 years old) was used that included subsets of patients that were GC-naïve at entry and remained naïve during follow up visits (n = 8), patients that were naïve at entry then went onto daily GC regimen during follow up visits (n = 10) and patients that were treated at entry and remained treated during follow up visits (n = 13). Of the 10 pre and post treated DMD patients above, 4 had more than one time point without GC treatment before receiving treatment while the remaining had only one naïve time point before going onto treatment. For the cross-sectional analysis, we used one unique sample from the subjects who were GC-naïve at entry (n = 18) and age matched healthy volunteers (n = 12) and screened levels of 1,310 unique proteins using SomaScan platform as described in the method. By focusing on GC-naïve DMD, we confirmed several previously identified biomarkers^1–3^ and also identified novel biomarkers associated with DMD. In this new study, levels of 108 proteins were significantly elevated while 70 proteins were significantly lower in sera samples of GC-naïve DMD patients compared to age matched healthy controls with a p-value adjusted for multiple testing <0.05 (Supplemental Table S1). As depicted in Fig. 1, the greatest segment of elevated biomarkers (~40%) included muscle centric proteins (e.g. thin filament proteins, glycolytic enzymes, calcium binding proteins and other muscle specific enzymes), but also included some pro-inflammatory factors such as C-C motif chemokines 2 and 18 (CCL2, CCL18), C-X-C motif chemokine 10 (CXCL 10), Interleukin–6 (IL6), and angiopoietin-2 (ANGPT2), as well as some peptidases and proteases such as aminoacylase-1 (ACY1), leukotriene A-4 hydrolase (LTA4H), calpain I (CAPN1), dCTP pyrophosphatase 1 (DCTPP1), methionine aminopeptidase 2 (METAP2), Xaa–Pro aminopeptidase 1 (XPNPEP1), and ubiquitin carboxyl–terminal hydrolase 25 (USP25)We identified three myogenesis regulating factors CDO (BOC), disintegrin and metalloproteinase domain–containing protein 12 (ADAM12) and cysteine and glycine-rich protein 3 (CSRP3) as being significantly elevated in the young DMD boys compared to age matched controls.

Interestingly, the majority of biomarkers that were decreased in GC-naïve DMD relative to controls were of cell adhesion origin (e.g. cadherins-3 and -5, contactin-4, osteomodulin, neural cell adhesion molecule L1-like protein, and von Willebrand factor), several growth/cell differentiation regulating factors such as Ret, GDF11/8, circulating growth hormone receptor (GHBP), BDNF/NT–3 growth factors receptor, insulin-like growth factor binding proteins-3 and 5 (IGFB3 and IGFBP5) and circulating epidermal growth factor receptor, innate immune regulators such as CD59 glycoprotein (CD59), complement decay-accelerating factor (DAF), immunoglobulin D (IGHD), complement component C8 (C8), complement C1q subcomponent and a few other proteins with different functions such as beta-Ala-His dipeptidase (CNDP1), gelsolin (GSN), circulating advanced glycosylation end product-specific receptor (AGER) and osteopontin (SPP1).

**Figure 2:**
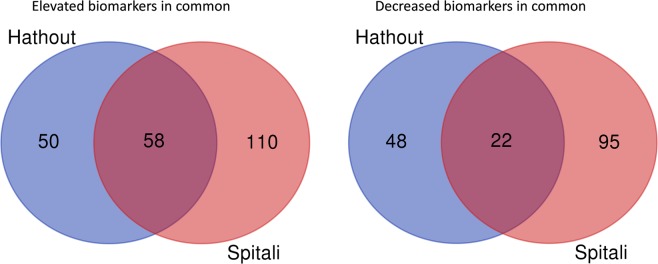
Pie chart showing the numbers of overlapping and non-overlapping serum protein biomarkers between our study and the most recent study^3^ that used the same SomaScan approach but on a different DMD and control cohort.

Comparison of the data in this study to the most recent DMD biomarker data generated on an independent cohort of DMD and controls using the same SomaScan technology^3^ showed an overlap in 58 elevated and 22 decreased serum proteins in DMD relative to controls (Fig. 2, Supplemental Table S2). However, a large number of the elevated proteins in DMD, 50 from our study and 110 from the previous study^3^, as well as a large number of decreased proteins in DMD, 48 from our study and 95 from the previous study^3^ did not overlap.

### Association of circulating DMD protein biomarkers with age

**Figure 3:**
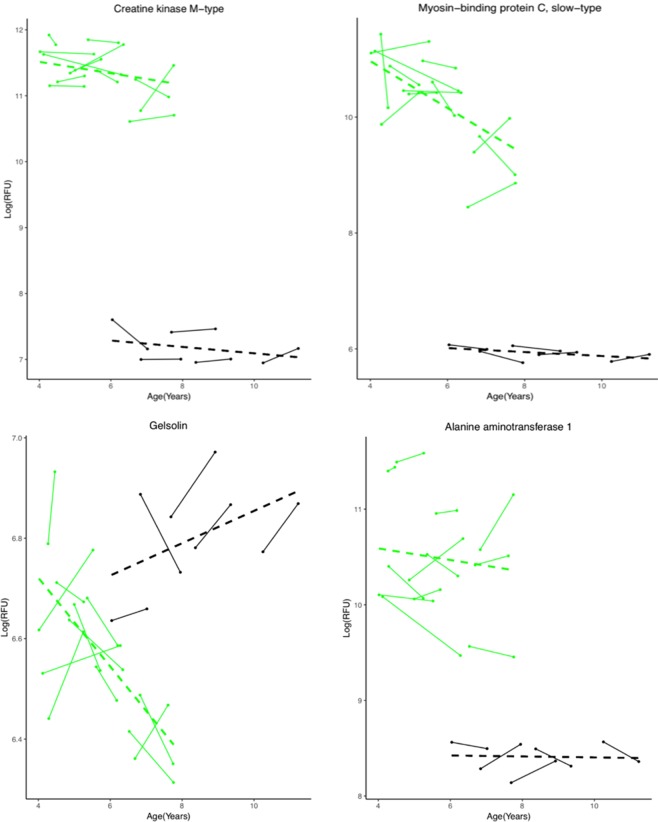
Longitudinal trajectory of selected examples of DMD serum protein biomarkers and their association with age. CK-M remained relatively stable over time in young GC-naïve DMD patients (p = 0.506) and in controls (p = 0.622) within the age range studied. Myosin-binding protein C declined over time in DMD (p = 0.0477) and remained unchanged over time in controls. Gelsolin neared significance in its association with age (p = 0.0598); it decreased over time in untreated DMD patients while slightly increasing with age in controls. Alanine aminotransferase-1 while elevated in DMD relative to control remained stable over time in both DMD (p = 0.607) and controls. Green lines are DMD patients (n = 12) and black lines are healthy controls (n = 5). The dashed line represent the regression line in each group. P values are adjusted for multiple testing.

Age has been previously identified as a major confounding variable on the concentrations of serum protein biomarkers in DMD patients^2,3^. Depending on the age range studied, a biomarker could be present at different concentrations in DMD relative to controls. The majority of blood circulating biomarkers are elevated in DMD relative to control at younger age then decline overtime and reflect loss of muscle mass. As mentioned above, in this cohort of 31 young DMD patients used in this study, we identified subsets of DMD patients that were GC-naïve at entry and remained naïve during the follow up visits (n = 8), patients that were naïve at entry then went onto daily GC regimen during the follow up visits (n = 10). Of these 10 pre and post treated DMD patients above, 4 had more than one time point without GC treatment before receiving treatment while the remaining had only one naïve time point before going onto treatment. For the association with age analysis, we used all available time points for 12 GC-naïve DMD subjects (8 + 4) to increase statistical power. A linear mixed effect model was used to investigate the association of the 178 identified DMD biomarkers above with age (i.e., over time). In this subset of DMD patients, serum blood samples were collected over approximately 1 year, on average with a minimum follow-up range of 0.19 to a maximum of 2.16 years. After adjusting for multiple testing, only three biomarkers, coiled–coil domain–containing protein 80 (CCDC80), mitogen-activated protein kinase 14 (MAPK14) and myosin-binding protein C, slow-type (MYBPC1) were found to change in their concentrations over time (p value ≤ 0.05). Additional biomarkers including heat shock protein HSP90-alpha/beta, heat shock 70 kDa protein 1A (HSPA1A), integrin alpha-I: beta-1 complex (ITGA1:B1), Glycogen synthase kinase-3 alpha/beta (GSK3a/b) and gelsolin (GSN) neared significance in their association with age (p < 0.06, after adjusting for multiple testing). The circulating levels of these biomarkers decreased longitudinally in DMD patients while their trend remained relatively unchanged in controls (Supplemental Table S1). MAPK14, MYBPC1 HSP90, HSPA1A, and GSK3a/b are muscle centric proteins and their decline might reflect early loss of muscle fiber integrity. CCDC80 is an extracellular protein; it was elevated at young age in DMD relative to controls and rapidly declined overtime. Gelsolin (GSN) circulated at low concentrations in sera of GC-naïve DMD relative to controls then further decreased with age in DMD while it remained unchanged or slightly increased with age in controls. Other muscle centric biomarkers, such as CK-M, carbonic anhydrase III (CA3), troponin I (TNNI3), calcium/calmodulin-dependent protein kinase type II subunit alpha and beta (CAMK2A and CAMK2B), mitogen-activated protein kinase 12 (MAPK12), malate dehydrogenase cytoplasmic (MDH1) that were previously shown to be affected by age when looking at a wider age interval (4 to 28 years old)^2,3^ remained relatively stable over time within this narrower age window selected in this study (4 to 7 years old). Several other biomarkers although differentially altered in their concentrations between DMD and controls did not significantly change in their concentrations over time (e.g. alanine aminotransferase 1). These biomarkers that were found stable in their concentrations overtime could be useful pharmacodynamic biomarker candidates to assess different therapies in DMD boys between the age of 4 to 7 years old. Figure 3 shows examples of the different type of serum protein biomarkers described above and their association with age.

### Glucocorticoid responsive serum protein biomarkers in DMD

**Figure 4:**
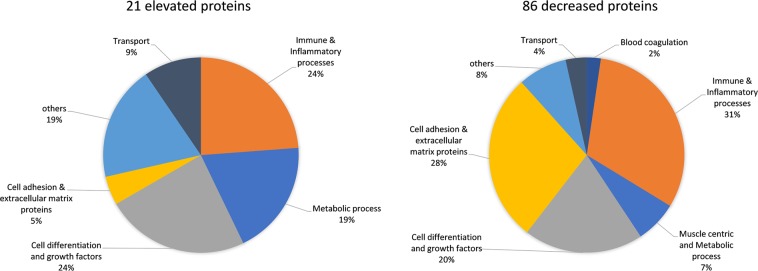
Pie chart depicting the biological process and molecular function of pharmacodynamic biomarkers that were increased (21 total) and those that were decreased (86 total) in DMD patients after treatment with GC.

In this cohort, we identified subsets of DMD patients that were GC-naïve at entry and remained naïve during follow up visits (n = 8), patients that were naïve at entry but then were treated with GCs during follow up visits (n = 10), patients that were treated at entry and remained treated during follow up visits (n = 13) as well as sera samples from 12 age matched healthy controls. In this new analysis, we confirmed our previously identified GC-responsive pharmacodynamic (PD) biomarkers^14^ but also discovered novel biomarkers that responded to GC treatment. Focusing on the pre- and post-GC treated group, we identified 86 proteins that were significantly decreased and 21 proteins that were significantly increased (adjusted p value < 0.05, paired t-test) by GC treatment (Supplemental Table S3). Figure 4 shows the grouping of these GC serum protein PD biomarkers by their cellular processes and function.

**Figure 5:**
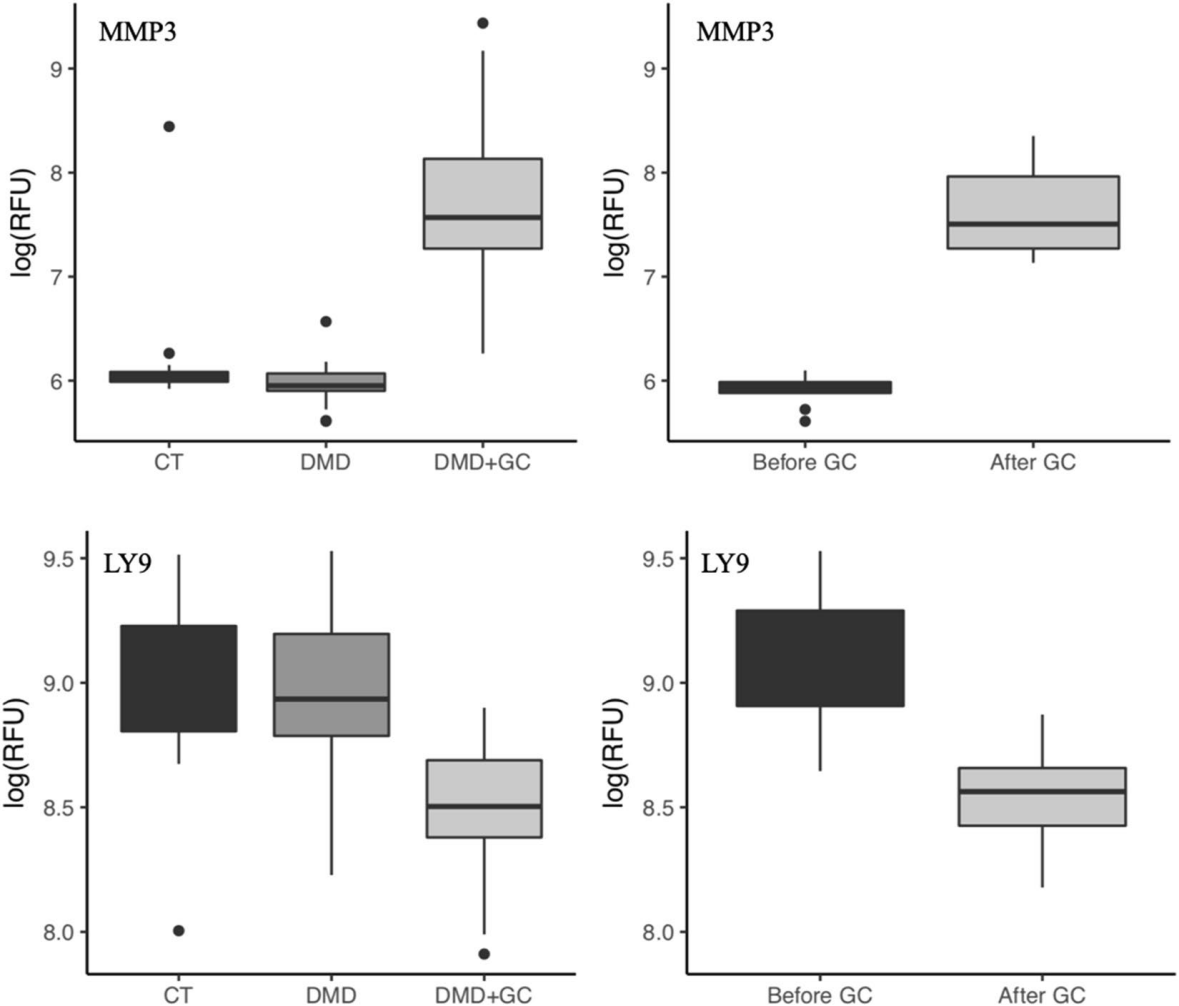
Representative exemplar of serum proteins that are not associated with DMD but responded to GC treatment. Left panel shows examples for Ly-9 and MMP3 in cross-sectional analysis using healthy controls (CT, n= 12), GC-naïve DMD patients (DMD, n = 18) and GC treated DMD patients (DMD + GC, n = 31). Right panel shows these same biomarkers in pre and post GC treated DMD patients (n = 10) using paired t-test. P values were adjusted for multiple testing in both cross-sectional and longitudinal analysis (***p < 0.001, **p = 0.016).

Of the 107 serum proteins that were affected by GC use in DMD, only 27 were associated with DMD while the remaining 80 were not significantly different in their concentrations between GC-naïve DMD and controls at baseline. Mostly lymphocyte- and monocyte-associated proteins decreased following GC treatment and reflect immune suppression. GC also caused a decrease in the concentrations of a number of proteins associated with cell adhesion, extracellular matrix and bone development which might reflect side effects of GC. Other proteins that were not significantly altered in their concentrations between the GC-naïve DMD group and controls and were significantly increased by GC consisted of proteins associated with metabolic processes, growth factor binding proteins and extracellular proteases and could also be associated with side effects of GC. Examples of such biomarkers that are purely associated with GC use are shown in Fig. 5 for T-lymphocyte surface antigen Ly-9 (LY9) protein that is associated with immune cells and MMP3 that is involved in extracellular matrix degradation.

**Figure 6:**
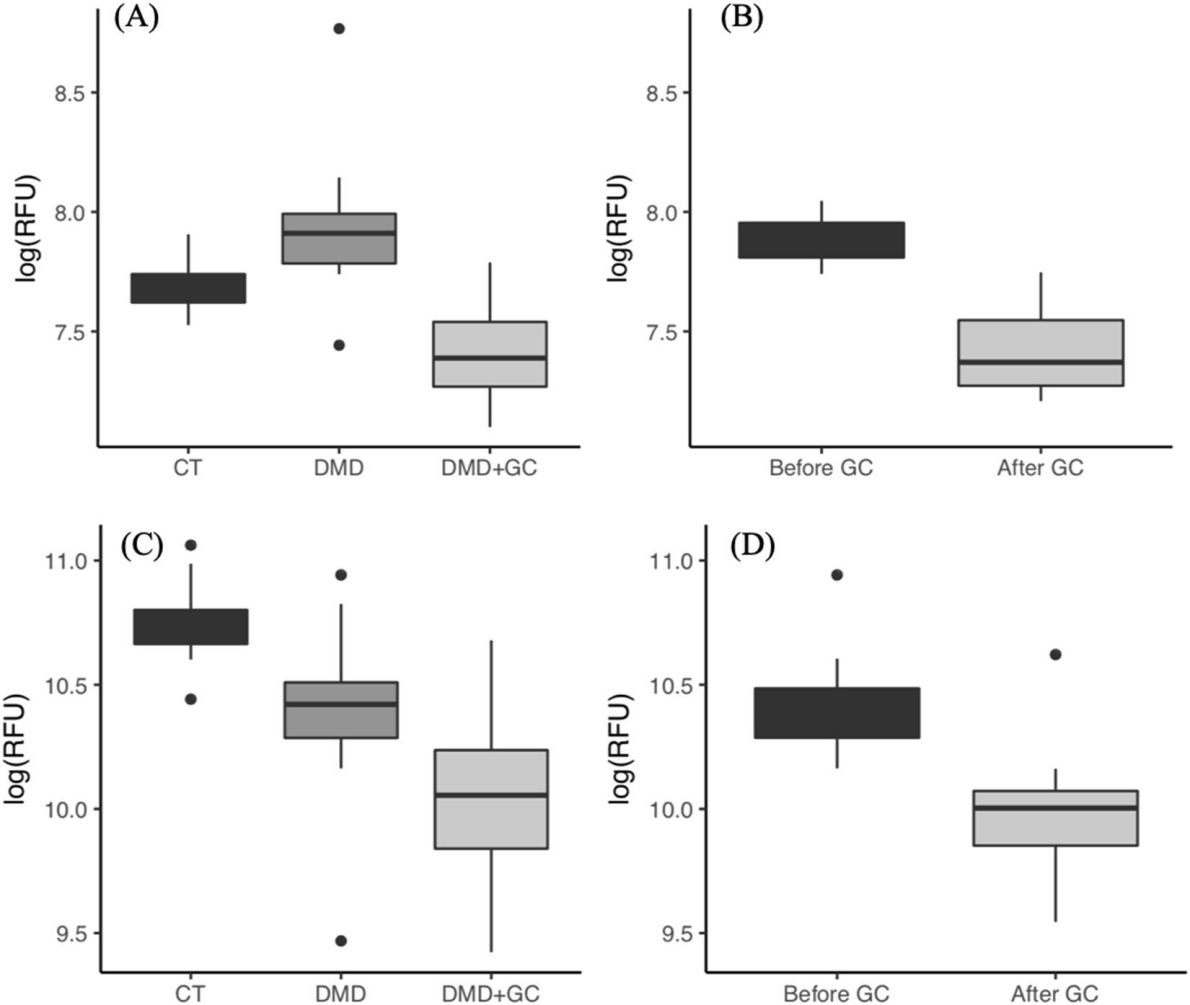
Representative exemplar of DMD associated biomarkers that responded to GC treatment. Left column shows cross-sectional comparison of the biomarker concentrations in healthy (CT) controls (n = 12), GC-naïve DMD (n = 18), GG treated DMD (n = 31), p values are adjusted for multiple testing. Right panel shows paired t-test analysis of the same biomarker in pre and post GC treated DMD patients (n = 10). (**A**,**B**) Lumican was elevated in GC-naïve DMD patients then decreased following GC treatment (****p < 0.001, ***p < 0.02). (**C**,**D**) Osteomodulin was slightly lower in GC-naïve DMD patients relative to controls and further decreased following GC treatment (**p = 0.015, *p < 0.05).

The 27 biomarkers that were initially altered in their concentrations in GC-naïve DMD patients relative to controls and then responded to GC treatment could be considered as candidate of disease specific GC PD biomarkers. After investigating the association of age, GC use, and their possible interaction on these 27 biomarkers using linear mixed effect models, 17 were retained as DMD associated biomarkers and GC-responsive and these are listed in Table 1 with corrected p-values for age, GC use and the interaction between the two (adjusted p-value for interaction between age and GC use). One biomarker, CCDC80 was found associated with both GC use and age (p interaction = 0.024) while the remaining 16 biomarkers were associated with GC treatment. Biomarkers that were initially elevated in GC-naïve DMD group relative to the control group and then decreased following GC treatment included CCD80, IGFB2, BMP6, lumican (LUM), angiopoietin-2 (ANGP2), fibrinogens (FGA, FGB and FGG), disintegrin and metalloproteinase domain-containing protein 12 (ADAM12), chordin-like protein 1 (CRDL1) and sex hormone–binding globulin (SHBG). Biomarkers that were initially decreased in GC-naïve DMD patients relative to control then increased following GC treatment included angiotensinogen (ANGT), beta-Ala-His dipeptidase (CNDP1), apolipoprotein L1 (ApoL1) and insulin-like growth factor-binding protein 5 (IGFB5). Finally, another class of proteins such as contactin-4 (CNTN4), osteomodulin (OMD) and advanced glycosylation end product-specific receptor (AGER) were initially decreased in GC-naïve DMD patients relative to controls but further decreased following GC treatment. Examples of these different categories of DMD associated biomarkers that responded to GC treatment are shown in Fig. 6 with their serum levels in controls versus GC-naïve DMD patients and GC treated DMD patients as well as in pre and post treated DMD patients.

**Table 1:** List of DMD specific biomarkers that responded to GC treatment.

Uniprot_ID	Abbreviated protein name	P_age_adj	P_GC_adj	P_interaction_adj	Conclusion
Q76M96	CCDC80	0.008	0.015	0.024	Both
Q96KN2	CNDP1	0.584	<0.001	0.161	Only GC
P01019	ANGT	0.545	<0.001	0.170	Only GC
Q9BU40	CRDL1	0.100	0.0187	0.161	Only GC
P04278	SHBG	0.582	0.002	0.475	Only GC
P18065	IGFBP2	0.335	0 < 0.001	0.628	Only GC
Q8IWV2	CNTN4	0.133	0.001	0.170	Only GC
P22004	BMP6	0.100	0.031	0.272	Only GC
P02671 P02675 P02679	FGA, FGB, FGG	0.090	0.0357	0.475	Only GC
P24593	IGFB5	0.147	<0.001	0.715	Only GC
Q15109	AGER	0.545	0.001	0.628	Only GC
Q99983	OMD	0.544	0.0014	0.627	Only GC
Q5KU26	COL12	0.268	0.009	0.627	Only GC
P51884	LUM	0.190	<0.001	0.475	Only GC
O43184	ADAM12	0.167	0.005	0.647	Only GC
O14791	ApoL1	0.464	<0.001	0.751	Only GC
O15123	ANGP2	0.263	<0.001	0.810	Only GC
P17936	IGFB3	0.090	<0.001	0.847	Only GC

All p values are adjusted for multiple testing with corrections applied to the three families of regression coefficients.

In summary, we identified two major classes of GC PD biomarkers in DMD patients. First, GC PD biomarkers that were not different in their concentrations in untreated DMD patients versus healthy controls but either decreased or increased following GC treatment. These biomarkers are purely associated with GC use and reflect immune suppression and possibly side effects of GC. A second group of GC PD biomarkers consisted of those that were different in their concentrations in untreated DMD patients versus controls but responded to GC treatment and tend to return to the concentrations seen in healthy controls except for OMD, CNTN4 and AGER that were decreased in their concentrations in DMD patients relative to controls at baseline then further decreased following GC treatment. These DMD associated biomarkers that responded to GC treatment could reflect true GC efficacy markers in DMD and they are discussed further below.

## Discussion

Blood circulating biomarkers are becoming increasingly attractive to use as tools to assess disease progression and predict response to therapies in DMD^14,16^. Because they can be easily accessed and are less subjective than physical outcome measures, they can potentially be integrated into clinical trials as primary and/or secondary outcomes.

About 45% of the identified biomarkers herein, including their directions (e.g. elevated or decreased in DMD patients vs controls), confirmed previously identified biomarkers^1–3,17^. Here, we also identify additional biomarkers and differentiate those that are DMD-associated from those that are associated with GC use. Comparison of our study (using GC-naïve DMD patients and the same SomaScan technology) to the most recent study by Spitali *et al*.^3^ confirmed 82 serum protein biomarkers in common that were differentially altered in their concentrations in DMD patients versus controls. All of the elevated proteins were concordant between the two studies except for Leukotriene A-4 hydrolase (LTA4H), Ubiquitin carboxyl-terminal hydrolase isozyme L1 (UCHL1), cytoplasmic aspartate aminotransferase (GOT1), Bone morphogenetic protein 6 (BMP6), and E3 ubiquitin-protein ligase CHIP (STUB1) which went in the opposite direction, elevated in our study and decreased in the earlier study^3^. All the overlapping biomarkers that were decreased in DMD patients relative to control between the two studies were concordant except for trefoil factor 1 and IGFBP5 that we show to be decreased in DMD patients relative to healthy controls while the earlier study showed that they were elevated in DMD patients relative to controls. Additionally, a large number of the elevated proteins in DMD patients compared controls, 50 in our study and 110 in previous study^3^, as well as a large number of the decreased proteins in DMD patients compared to controls (48 in our study and 95 in previous study) did not overlap. These differences between the two studies could be due to a combination of possibilities including random subject to subject variability, variability of SomaScan assay from run to run, difference in statistical methods used to analyze the data and the difference in age range and GC use between the two cohorts studied. For instance, in our study, the GC-naïve DMD boys were never treated with GC and their age ranged from 4 to 10 years old while in the previous study^3^, most of the patients were treated with GC and their age ranged from 5 to 15 years old. In the Results section, we show that GCs affected the concentrations of a significant number of serum circulating proteins that were not associated with DMD pathogenesis per se. Hence, GC use should be taken into consideration when validating DMD biomarkers.

We found that current databases and tools for classification of large omics data to be biased toward cancer related research. Hence, manual grouping of the biomarkers using curated Uniprot database was utilized in classifying the list of biomarkers based on their tissue of origin, function, and cellular localization. Rather than discussing the potential significance of each identified biomarker individually, we classified them into groups based on putative origin and function, as follows.

### Muscle injury biomarkers

Muscle associated proteins including structural proteins, glycolytic enzymes and some other muscle enzymes were by far the most significantly elevated circulating biomarkers in young GC-naïve DMD patients compared to healthy controls. Most of these biomarkers reflect sarcolemma instability, muscle fiber leakage and behaved like CK. These CK like biomarkers were previously reported by others across different labs using independent cohorts^1–3,17^ and will not be further discussed in detail herein. It is however important to point out that some of these muscle injury biomarkers declined overtime even within a narrow age range 4–10 years old and might reflect early loss of muscle mass that is often not apparent at this young age using other methods such as MRI and/or muscle biopsies. However, other muscle injury biomarkers (CKM, TNNI3, CAMK2A, CAMK2B, MAPK12, MDH1 and GP1) remained stable overtime in this narrow age range studied. These muscle injury biomarkers could be useful PD biomarker candidates to demonstrate efficacy of dystrophin replacement therapies and other sarcolemma stabilizing therapies in younger DMD patients. Indeed, a recent study conducted by our group, showed a significant decrease in CK activity from baseline after two weeks of treatment in young DMD patients with the new dissociative steroidal drug vamorolone^18^. Similarly in this study, although it did not reach significance after adjusting for multiple testing (p = 0.099), CK-M also decreased from baseline in DMD patients following treatment with GCs (see Supplemental Table S1). The decline in CK-M was not associated with age in the narrow age range studied (p = 0.50 with adjusted multiple testing, see Supplemental Table S1).

### Biomarkers associated with myogenesis and muscle development

Three myogenic biomarkers were found to be significantly elevated in their concentrations in GC-naïve DMD relative to controls (e.g. ADAM12, BOC and CSRP3). ADAM12 was the only marker in this category that responded to GC treatment in DMD patients and decreased toward the concentrations seen in healthy controls following treatment. ADAM12 exists as a membrane bound form but also as a soluble circulating form. The circulating form has been shown to promote myogenesis *in vivo*^19^. BOC and CSRP3 are cell surface proteins that have been reported to regulate myogenesis via cell-cell contact^20–23^. The abundance of these myogenic promoting factors in sera samples of young DMD patients might reflect the active muscle regeneration occurring at an early stage of the disease. The significance of the reduction of circulating ADAM12 by GC treatment is not well understood at this time and further experiments are needed to explain the physiological significance of this decrease.

Other markers involved in muscle development that were also found to be different in their concentrations in GC-naïve DMD patients relative to controls are GDF11 and GDF8. These were detected at lower concentrations in DMD patients compared to controls and slightly decreased with age in GC-naïve DMD patients but were observed to increase with age in healthy controls. The decrease in the concentration of circulating GDF8, also known as myostatin, in DMD patients relative to controls is in agreement with previously reported ELISA data in DMD patients relative to healthy controls^24^. The decrease in concentrations of this circulating marker might be associated with the classical calf hypertrophy seen in DMD boys^25,26^ but the physiological significance of this decrease remains to be studied.

### Peptidase and protease biomarkers

Concentrations of some peptidases, proteases and proteasome associated enzymes (e.g. methionine aminopeptidase 2, calpain-1, ubiquitin carboxyl-terminal hydrolase 25 (USP25), aminoacylase-1, dCTP pyrophosphatase 1, 26S proteasome non-ATPase regulatory subunit 7, Xaa-Pro aminopeptidase 1, proteasome subunit alpha type-1) and few other hydrolases were found to be significantly elevated in GC-naïve DMD patients relative to controls at baseline, remained relatively stable overtime within the age range studied and did not respond to GC treatment. The high concentrations of these peptidases and proteases in DMD is most likely associated with the drastic protein turnover of skeletal muscle that occurs during myofiber degeneration and regeneration at an early stage of the disease^27,28^. This is supported by the numerous studies showing that calpain activity is higher in dystrophin deficient skeletal muscle compared to healthy muscle (for more detail, see review by Hollinger & Selsby^29^). The elevated concentration of calpain-I, in our study, could be caused by an increase in calcium influx during membrane disruption, as evidenced by a correlation between increased calcium influx and elevated concentration of calpain in muscle from DMD patients and mdx mice^30^. Calpains degrade myofibrillar and sarcomeric proteins such as titin whose fragments were also found to be significantly elevated in the blood circulation and in urine of DMD patients at younger ages^17,31,32^. Unfortunately, targeting inhibition of calpains as a potential therapy for DMD failed to restore muscle health in a dystrophin deficient canine model^33^, possibly because the increase in calpain expression is secondary to rather than a cause of the pathogenesis. The increase in circulating USP25 in DMD is novel in this study and might also be associated with the increased muscle protein turnover in DMD. Indeed, an earlier study^34^ has shown that *USP25* gene encodes three isoforms and one of them is a muscle specific isoform that regulates the turnover of myosin-binding protein C which was also found to be highly elevated in blood of DMD patients compared to controls in this study (see Supplemental Table S1). However, careful examination of the expression levels of these peptidases and proteases in dystrophin deficient skeletal muscle compared to normal muscle is needed to explore whether their increased levels in serum is simply due to muscle leakage.

CNDP1, also known as carnosinase, hydrolyzes carnosine, is another hydrolase that was found to be lower in serum of GC-naïve DMD patients relative to controls then significantly increased following GC treatment, in some cases exceeding the levels in controls. CNDP1, is a metalloprotease that degrades carnosine, a metabolite mainly found in skeletal muscle that regulates intracellular pH of muscle fibers and plays an important role during exercise^35^. Carnosine acts as an antioxidant and reduces free radicals generated from excess lipid and sugar oxidation^36^. Untargeted metabolomic analysis of skeletal muscle revealed lower concentrations of carnosine in a dystrophin deficient canine model compared to healthy controls that might explain the oxidative stress that is associated with DMD^37^. The significance of lower concentrations of circulating CNDP1 in GC-naïve DMD patients that increased following GC treatment at levels exceeding that in the controls is not well understood. But further studies correlating concentrations of circulating CNDP1 and carnosine in skeletal muscle are needed to define the significance of circulating CNDP1 as a biomarker for DMD.

### Cell adhesion and extracellular matrix biomarkers

Another class of biomarkers that were identified in this study is extracellular proteins. We observed significantly lower serum concentrations of proteins involved in cell adhesion, proteins regulating cell differentiation and growth and other extracellular proteins with miscellaneous function such as scavenger of advanced glycation end products (AGER) in GC-naïve DMD patients compared to healthy controls. These proteins are important in maintaining the integrity and structure of the extracellular matrix and alteration in their circulating concentrations might reflect the perturbed extracellular environment of dystrophic muscle fibers. Several of these adhesion and extracellular proteins were further affected in their concentrations by GC treatment. For example, OMD, AGER, cadherin-5 and contactin-4 were already decreased in DMD patients compared to controls at baseline but further decreased following GC treatment. The physiological significance of this GC induced decrease in concentrations of circulating adhesion proteins is not understood at present but further studies are needed to elucidate their role in DMD muscle pathogenesis and the significance of their decrease by GC. LUM on the other hand was elevated in GC-naïve DMD patients relative to healthy controls and returned to normal concentrations following GC treatment. LUM was previously shown to be elevated in fibrotic DMD skeletal muscle compared to healthy muscle^38^ and reduction by GC treatment could reflect an effect of GC on fibrosis in DMD.

### Pro-inflammatory biomarkers and complement factors

By focusing on young GC-naïve DMD patients, we were able to identify a useful set of pro-inflammatory biomarkers that were significantly elevated in untreated DMD relative to controls (e.g. FGG, IL-6, CXCL10, CCL18, CCL2, ANGPT2, TNFRSF1A, COL12, C5b-C6 complex). Interestingly, most of these circulating pro-inflammatory biomarkers were only moderately elevated (~2 fold) in GC-naïve DMD patients relative to controls and did not respond to GC treatment. Exceptions to this pattern included FGG, ANGPT2, and COL12; these normalized in DMD patients following GC treatment toward the levels seen in healthy controls. These data suggest that circulating pro-inflammatory biomarkers are at lower levels in DMD patients as compared to other pediatric inflammatory diseases such as juvenile dermatomyositis (JDM) where it has been shown that circulating levels of a subset of these same inflammation-associated biomarkers such as CXCL10, CCL2, and IL-6 were 10 to 100 fold higher in juvenile dermatomyositis patients compared to healthy controls^39^. Lower concentrations of circulating inflammation biomarkers in DMD have many causes and may be influenced by the variations in inflammation between different areas of the same skeletal muscle of a DMD patient. In contrast, inflammation in skin and muscle of JDM patients may be more uniformly present throughout the tissues.

On the other hand, the circulating concentrations of two complement inhibitors, complement decay accelerating factor (DAF), and CD59 were found to be lower in GC-naïve DMD patients compared to controls and this could be associated with involvement of membrane attack complex (MAC) in DMD^40^. However, further analysis of the concentrations of CD59 and DAF in skeletal muscle biopsies from DMD patients and heathy controls will be needed to confirm the involvement of complement attack in DMD pathogenesis.

### Other miscellaneous biomarkers

Several identified biomarkers in DMD did not belong to a specific group but were found to be significantly altered in their concentrations in GC-naïve DMD patients compared to controls and might be of relevance in the pathogenesis of dystrophin-deficient muscle. The majority of these biomarkers were lower in GC-naïve DMD patients relative to controls and either remained lower across the age span studied or further decreased with age or GC use (e.g. gelsolin, adiponectin, AGER). For example, plasma gelsolin, an important protein in the scavenging and clearing of actin bundles released by damaged cells into the circulation was found to be decreased in GC-naïve DMD patients relative to controls at baseline and further decreased with age in untreated DMD patients. This is consistent with previous reports showing lower concentrations of circulating gelsolin in DMD patients compared to controls^2,3,41^. The protein showed a steady decline with age in untreated DMD patients while it remained constant or slightly increased with age in healthy controls. GC treatment tends to stop the decline of this protein overtime. The lower levels of circulating gelsolin in DMD patients relative to controls is not well understood at this time but we speculate that gelsolin might be sequestered around degenerating skeletal muscle to help clear actin filament shedding. Further analysis of gelsolin concentrations in serum samples and skeletal muscle will be needed to test this hypothesis.

Adiponectin is another circulating biomarker of interest and behaved in a similar fashion to gelsolin. Adiponectin levels were low in sera of untreated young DMD compared to controls and slightly decreased, although not significantly, with age in DMD patients while it remained unchanged in controls. GC treatment appeared to stabilize this decline. We and others have previously shown that circulating adiponectin is also low in mdx mouse model compared to wild type mouse^17,42^. Furthermore, adiponectin was shown to protect skeletal muscle from inflammation/injury and improve myogenesis and muscle force in the mdx mouse model^42,43^.

One important finding in this study is that treatment of DMD patients with GC affected the levels of a large number of serum proteins that were not associated with the disease per se (80 proteins). This reflects the broader effect of GC on gene transcription^44^. In contrast, GC normalized only a small subset of serum protein biomarkers that were associated with the disease (17 out of 178 proteins) indicating the persistence of disease activity despite daily treatment with the drug. Although this is a small number of biomarkers, it is consistent with the physiologic effects of GCs in DMD. As discussed above, 3 of the 17 DMD biomarkers that were elevated in DMD patients relative to controls, and also responded to GC treatment, were linked by other studies to inflammation and the innate immune response (e.g. Fibrinogens, ANGPT2 and COL12). The decrease observed in these proteins by GC might reflect efficacy of GC treatment in DMD patients. Three proteins that were elevated in untreated DMD patients (LUM, ADAM12 and BMP6) were linked to fibrosis by other studies and their decrease by GC could also reflect a benefit of the treatment. The significance of normalization of the remaining 9 proteins by GC (CCDC80, CNDP1, ANGT, CRDL1, SHBG, IGFBP2, IGFBP3, IGFBP5, ApoL1) and further decrease in AGER, CNTN4 and OMD by GC remains unclear. Future studies using knockdown of specific genes in mouse models may increase insight into the physiological role of these proteins in DMD patients and also the significance of their response to GC treatment.

One limitation of the current study is the lack of serum and muscle biopsy samples from the same patients that could provide further insight into the levels of these biomarkers in muscle tissue at an important site of the ongoing pathological change in muscle. Such a study could help to select the best biomarker candidates for further study. Another important limitation of our study design is the inability to bridge the identified serum circulating biomarkers to clinical outcomes. The number of patients studied does not provide sufficient power to conclusively bridge the biomarkers identified in this report with strength and function outcomes that can predict clinical milestones^45^.

## Conclusion

In summary, a variety of circulating protein biomarkers were identified in this study reflecting the complex nature of DMD pathogenesis and the response to GC treatment. While this is important to understanding the molecular mechanism of DMD pathogenesis and to define novel therapeutic targets, these blood accessible biomarkers could prove useful to to DMD clinicians, researchers and patients as tools to assess disease progression and response to therapies. Hence, it is important to bridge these biomarkers to disease milestones and clinical outcomes in DMD in follow-up studies. With this refined catalogue of serum proteins for DMD, we hope to set the stage for future studies that will rely on serum biomarkers for the conduct of clinical trials and drug development programs in DMD.

## Materials and Methods

### Study participants and data/specimens collection

In this study, we used a subset of DMD patients, age range 4–10 years old (n = 31) and age matched healthy controls (n = 12) enrolled through the Cooperative International Neuromuscular Research Group-Duchenne Natural History Study (CINRG-DNHS)^46^. The study protocol was approved by Institutional Review Boards at all participating institutions that provided us with serum samples. These included, the Office of Research IRB administrations at the University of California Davis, Davis, CA, the University of Pittsburgh, Pittsburgh, PA, Children’s National Health System, Washington DC, the Conjoint Health Research Ethics Board, University of Calgary and the Human Subject Research Review Committee at Binghamton University, NY. All patients had standard DNA testing by their physician and were genetically confirmed to have DMD by the CINRG genetic counselor before enrolling them into the natural history biomarker study. Informed written consent was obtained from the parents of the participants or their legal guardians for biomarker studies at each site. All methods were performed in accordance with the relevant guidelines and regulations to protect human subjects and ensure that participants remain de-identified during samples analysis and data reporting. In this cohort of 31 young DMD patients (4–10 years old), we had subsets of DMD patients that were GC-naïve at entry and remained naïve during follow up visits (n = 8), patients that were naïve at entry then went onto daily GC regimen during follow up visits (n = 10) and patients that were treated at entry and remained treated during follow up visits (n = 13). Of the 10 pre and post treated DMD patients above, 4 had more than one time point without GC treatment before receiving treatment while the remaining had only one naïve time point before going onto treatment. Patients were typically prescribed either prednisone (0.75 mg/kg/day) or deflazacort (0.9 mg/kg/day). Detailed patient’s demographics including GC use are listed in Supplemental Table S4. Each DMD patient and 5 of the 12 controls had more than one visit with some DMD patients up to 4 visits to a CINRG study site during a 2 year follow up. Serum samples were prepared from each collected blood sample following a standardized operating protocol and stored in aliquots in polypropylene cryogenic Nalgene vials (Thermo Scientific) at −80 °C for subsequent biomarker analysis.

### Serum proteome profiling using SomaScan technology

Aliquots of 150 μl were prepared from each sample and submitted for proteome profiling using high throughput multiplexing aptamer based technology also known as SomaScan assay (Somalogic Inc., Boulder, CO). The principle of the SomaScan assay has been described in more detail elsewhere^47,48^ and briefly summarized herein. The SomaScan assay consists of a panel of protein-specific Slow Off-rate Modified DNA aptamers (SOMAmers) that bind to the target protein in its native folded from with high affinity and specificity. In this study, we used the 1.3 k SomaScan version consisting of 1,310 aptamers targeting 1,310 unique serum proteins. Each aptamer is tagged with short DNA sequence enabling high throughput quantification using custom hybridization array. Protein quantities are recorded as relative fluorescent units (RFU) from microarrays. All arrays were done using a dilution series of each sera sample so that the signal/noise ratio of each aptamer/protein pair was optimized.

### **Data proc**essing and statistical analysis

Raw data generated from the SomaScan assay was first hybridization control normalized and median signal normalized. Then, the normalized data was processed using SomaSuite software and exported as an excel sheet containing de-identified patient ID, age and GC use along with protein name and RFU value for each protein in each samples. RFU data was log transformed to make the distribution approximately normal (as is standard practice). First, we performed a cross-sectional analysis to identify serum protein biomarkers that are significantly different in their concentrations between GC-naïve DMD (never-treated with GC) with a unique data point from each patient (n = 18) and age matched healthy controls (n = 12). Multiple independent sample t-tests were performed to compare log(RFU) values of 1310 serum protein biomarkers between these two groups. Using random intercept, linear mixed effect models, we also investigated whether the change in protein concentrations (all available time points for 12 GC-naïve DMD and 5 controls) is associated with age in naive patients and healthy controls. Note that some of the models did not converge due to numerical optimization issues (probably due to small sample size).

Next, we focused on the DMD patients that were naïve at entry but then were treated with GC during the follow up visits (n = 10) to identify serum protein biomarkers that were significantly impacted by the GC use. For each protein, we performed a paired t-test to compare each patient’s log(RFU) concentrations measured at the last visit before the GC treatment and those measured at the end of the study. For both the two independent sample and paired t-tests conducted, their p-values were adjusted using the Benjamini-Hochberg procedure for multiple testing^49^.

We then focused on the subset of proteins that were found to be both significantly different in GC-naïve DMD patients vs. controls and were responsive to GC in a paired analysis. For the longitudinal analysis, random intercept, linear mixed effect models were used to investigate the association of age, GC use, and their possible interaction on the (natural log) of protein RFUs in subjects with repeated measurements (all 31 DMD subjects with at least 2 time points for each). For most subjects, 4 time points were used (for some, only two time points were used). Note that the age coefficient represents the change in protein concentrations, on average, associated with a one unit increase in age (centered) for naïve subjects. The GC-use coefficient represents the average difference in mean protein concentrations between GC-treated and naive patients for a patient of average age. The coefficient for the interaction represents the average difference in slope of age (centered) on biomarker levels between naïve and GC-treated subjects, and so, a significant interaction indicates that a 1-unit change in age is associated with different amounts of change in the protein concentrations in naïve and GC-treated subjects, on average. If the interaction coefficient was non-significant, we looked at the main effects to form our conclusion, i.e., whether the change in protein concentrations was associated with age only or GC-use only. All statistical analyses were conducted using *R*^50^. Linear mixed effect models were run using the *lme4* and *lmerTest* packages^51^.

## Supplementary information

Supplemental Table S1

Supplemental Table S2

Supplemental Table S3

Supplemental Table S4
